# Seismic inversion with L_2,0_-norm joint-sparse constraint on multi-trace impedance model

**DOI:** 10.1038/s41598-022-26488-1

**Published:** 2022-12-17

**Authors:** Ronghuo Dai, Jun Yang

**Affiliations:** 1 School of Mathematics and Information, China West Normal University, Nanchong, Sichuan Province People’s Republic of China; 2 School of Mathematics, Zunyi Normal University, Zunyi, Guizhou Province People’s Republic of China; 3 School of Geoscience and Technology, Southwest Petroleum University, Chengdu, Sichuan Province People’s Republic of China

**Keywords:** Solid Earth sciences, Geophysics

## Abstract

Impedance inversion of post-stack seismic data is a key technology in reservoir prediction and characterization. Compared to the common used single-trace impedance inversion, multi-trace impedance simultaneous inversion has many advantages. For example, it can take lateral regularization constraint to improve the lateral stability and resolution. We propose to use the L_2,0_-norm of multi-trace impedance model as a regularization constraint in multi-trace impedance inversion in this paper. L_2,0_-norm is a joint-sparse measure, which can not only measure the conventional vertical sparsity with L_0_-norm in vertical direction, but also measure the lateral continuity with L_2_-norm in lateral direction. Then, we use a split Bregman iteration strategy to solve the L_2,0_-norm joint-sparse constrained objective function. Next, we use a 2D numerical model and a real seismic data section to test the efficacy of the proposed method. The results show that the inverted impedance from the L_2,0_-norm constraint inversion has higher lateral stability and resolution compared to the inverted impedance from the conventional sparse constraint impedance inversion.

## Introduction

In exploration geophysics, seismic inversion can infer out the elastic parameters or petrophysical properties of the underground formation from seismic data, especially seismic reflection data^1,2^.

The seismic inversion problem began to be dealt with deterministic methods, which are optimization procedures seeking the minimization of an objective function, normally the mismatch between the synthetic seismic that is obtained by perturbing an initial guess and the observed seismic reflection data^3^. In recent decades, seismic inversion has been successfully extended to a statistical framework for assessing the uncertainty of the inferred 3D subsurface elastic models^3^. Among statistical inversion, there are two different stochastic approaches. The first group of stochastic seismic inversion execute in a geostatistical iterative procedures, in which the model parameter is globally perturbed by stochastic sequential simulation algorithms^4–8^. The second group of stochastic seismic inversion is called Bayesian inversion methods, which is based on the solution of inverse problem using the Bayesian framework^3,9^.

From the type of reflection seismic data, seismic inversion contains the post-stack inversion^10^ and pre-stack inversion^11^. Impedance inversion of post-stack seismic data is one of the common used seismic inversion methods in oil and gas industry and is a key technology in reservoir prediction and characterization. The inverted impedance model has been wildly used in oil and gas reservoir prediction, reserve evaluation of oil and gas reservoirs, design of drilling location and trajectory, and so forth.

However, most geophysical inverse problems are ill-posed and the solution is extreme unstable. The best way to solve ill-posed inverse problem are performing regularization constraints^12^^.^ As a regularization option, the sparsity constraint of the inversion results has become more and more popular in the field of geophysics, especially among seismic inversion workers. It can not only achieve the goal of stable inversion solution, but also better describe the sparse characteristics of seismic data to improve the resolution of inversion results.

There are two forms to perform sparse regularization in the existing references. The one is synthesis prior form^13,14^. In geophysical inverse problems, it directly performs the sparsity constraint on the model parameters to be inverted. For example, the sparse spike inversion of post-stack seismic data directly uses the sparsity constraint on the reflectivity series^15^. Another option of sparse regularization is analysis prior form^14^. The analysis prior formulation assumes that the signal is not sparse but has a sparse representation in another basis (or transform). Unlike the synthesis prior formulation, it recovers the signal itself rather than the transform coefficients. For example, the total variation regularization in image processing is the L1-norm sparse constraint on the magnitudes of image’s gradient^16^. The total variation regularization has also been widely applied in geophysical inverse problems^17–20^. The minimum gradient support regularization in geophysical inversion is in fact the modified Cauchy prior sparse constraint on the magnitude of the model parameters’ gradient^12^.

In this paper, we deal with impedance inversion at the framework of deterministic inversion, which belongs to the category of the analysis prior formulation when the sparse regularization constraint has been used. The impedance inversion with sparse regularization constraint has been widely used in reservoir prediction and characterization and has achieved very successful practical effects. However, the sparse regularization in common used impedance inversion methods just constrains in time or depth direction (i.e. in vertical direction). In addition, it performs inversion through single trace by single trace. It cannot allow lateral regularization^18,21^. Hence, the lateral resolution and continuity is very coarse. To perform lateral regularization, we deal with seismic inversion in the multi-trace case and adopt L_2,0_-norm of multi-trace impedance model as an regularization constraint.

L_2,0_-norm is a joint-sparse measure or row-sparse^14^, which can not only measure the conventional sparsity with L_0_-norm in vertical direction, but also measure the lateral continuity with L_2_-norm in lateral direction. The joint-sparse or row-sparse has promoted the sparse signal representation and compressed sensing recovery^22,23^ and has been applied in many science fields, such as target detection^24^, color image restoration^25^, hyperspectral image processing^26^, art restoration^27^, feature extraction^28^, and applied geophysics^29^. Then, we use a split Bregman algorithm to solve the L_2,0_-norm joint-sparse constrained objective function. Next, we use a 2D numerical model and a real seismic data section to test the efficacy of the proposed inversion method. The results show that the inverted impedance from the L_2,0_-norm constraint inversion has higher lateral stability and resolution compared to the inverted impedance from the conventional sparse constraint impedance inversion.

## Methodology

### Joint-sparse

In the theory of sparse signal representation and compressed sensing recovery, the joint-sparse representation is proposed based on the similarity of multiple signals. It aims to reconstruct multiple unknown signals from a small number of observations that share a common observation operator and the same or similar support sets. In other words, when multiple signals have a joint sparse structure, their non-zero elements occupy the same or similar positions. If the multiple singles can be sparsely represented in some basis or dictionaries or through some sparse transforms, their sparse representation coefficients also share the same or similar support sets. If the multiple sparse representation coefficients are arranged in a matrix in columns, the matrix will become row-sparse^14^. Considered the observed data contains some noise, the problem of multiple signals’ joint-sparse recovery can be formulated as,

1 $$ \min ||{\mathbf{TX}}||_{2,0} ,s.t.||{\mathbf{A}} - {\mathbf{SX}}||_{F}^{2} \le \delta $$

where **S** is the observation operator, **X** is the matrix of multiple signals, **A** is the observed data matrix of multiple signals, **T** is the sparse transform operator, ||.||_*F*_ is the Frobenius norm of a matrix, *δ* is the level of observation errors. Here, ||.||_2,0_ is the L_2,0_-norm of a matrix; it is defined as the number of L_2_-norms of the rows. The “number of rows” acts as an outer L_0_-norm; it enforces minimizing the number of selected rows, thus enforcing row-sparsity. From the theory of sparse signal representation, all of the other sparse regularization options are different relaxations of L_0_-norm^13^. Hence, we choose L_0_-norm to measure the sparsity of rows. Of course, Eq. (1) belongs to the category of the analysis prior formulation.

### Impedance inversion with L_2,0_-norm joint-sparse constraint

#### Forward equation

The convolution model of seismic data is a simplification of the seismic acoustic field. The post-stack single-trace seismic data is considered to be the result of the convolution between the reflectivity series and a band-limited seismic source wavelet. For calculation, the convolution can be discretized as^30^ ,

2 $$ {\mathbf{d}}{ = }{\mathbf{Wr}} $$

where **d** is the vector of observed single seismic data trace, **W** is the wavelet convolution operator matrix, and **r** is the discrete reflectivities vector.

In seismic impedance inversion, the model parameters to be inverted are impedance. When the reflectivity is small, the relation between impedance and reflectivity series can be approximated as^31^,

3 $$ {\mathbf{r}}{ = }{\mathbf{Dm}} $$

where **D** is the first-order difference operator, *m*(*i*) is the *i*th element of vector **m**, and is defined as the logarithmic impedance.

Combine Eq. (2) with Eq. (3), we can obtain the forward equation for single trace seismic impedance inversion, i.e.,

4 $$ {\mathbf{d}} = {\mathbf{WDm}} = {\mathbf{Gm}} $$

where **G** = **WD** is the forward operator matrix.

In the case of multi-trace seismic impedance inversion, we simultaneously consider multiple seismic traces for inversion. Assume that there are *L* traces, i.e.,

5 $$ {\mathbf{B}} = {[}{\mathbf{d}}_{{1}} {,}{\mathbf{d}}_{{2}} {,}...{,}{\mathbf{d}}_{j} {,}...{,}{\mathbf{d}}_{L} {]} $$

6 $$ {\mathbf{Y}} = {[}{\mathbf{m}}_{{1}} {,}{\mathbf{m}}_{{2}} {,}...{,}{\mathbf{m}}_{j} {,}...{,}{\mathbf{m}}_{L} {]} $$

where **d**_*j*_ is the *j*th seismic data trace, **m**_*j*_ is the *j*th logarithmic impedance trace. Hence, **B** is the seismic data matrix including *L* seismic traces arranged in columns, **Y** is the logarithmic impedance matrix including *L* logarithmic impedance traces arranged in columns.

Hence, the forward equation for multi-trace impedance inversion is,

7 $$ {\mathbf{B}} = {\mathbf{GY}} $$

#### Objective function

In sparse constraint seismic inversion, we think the reflectivity series of underground formations are sparse. Hence, under the least square criterion to deal with the effects of random noise and the constraint of L0-norm spare regularization for reflectivity series, the objective function for sparse constraint multi-trace seismic impedance inversion is given by,

8 $$ \min f({\mathbf{Y}}) = ||{\mathbf{B}} - {\mathbf{GY}}||_{F}^{2} + \alpha ||{\mathbf{DY}}||_{0} $$

where *α* is the regularization parameter for sparse constraint. Here, ||.||_0_ is the L_0_-norm of a vector obtained from arranging all the elements of matrix **DY** starting with the first column.

Although the above objective function is constructed under the framework of multi-trace inversion, there is still no any lateral regularization constraint. It is because the conventional sparse regularization constraint only considers the vertical sparsity, and does not consider the lateral variation feature and continuity of multi-trace impedance model. Although the underground strata are not uniform in lateral direction, the lateral changes are relatively stable in sedimentary strata, i.e., the strata have local lateral continuity. The reflectivity series and impedance from near traces are local similar. Hence, we can think the multi-trace reflectivity series have local similar support sets. In other words, we can think the multi-trace impedance model can be local joint-sparsely represented through the first-order difference transforms, i.e. the matrix DY has joint-sparsity. Hence, we use L_2,0_-norm as an joint-sparse regularization constraint to replace L_0_-norm in Eq. (8) and construct the following the objective function for multi-trace seismic impedance inversion,

9 $$ \min f({\mathbf{Y}}) = ||{\mathbf{B}} - {\mathbf{GY}}||_{F}^{2} + \alpha ||{\mathbf{DY}}||_{2,0} $$

From joint-sparse, one can know that the L2,0-norm can not only measure the sparsity in the vertical direction, but also consider the lateral similarity and continuity of multi-trace impedance model to increase the lateral resolution and continuity.

Usually, the inversion can improve the overall stability through compensating the lacked low-frequency components in original seismic data^20^. To compensate the low frequency components which coincide with true geological background, a priori model can be used^17^. Adding the a priori model constraint into the objective function (9), it can be updated as,

10 $$ \min f({\mathbf{Y}}) = ||{\mathbf{B}} - {\mathbf{GY}}||_{F}^{2} + \alpha ||{\mathbf{DY}}||_{2,0} + \rho ||{\mathbf{Y - Y}}_{{{\text{prior}}}} ||_{F}^{2} $$

where *α* is the regularization parameter for a priori model constraint, **Y**_prior_ is the a priori multi-trace logarithmic impedance model.

#### Split Bregman algorithm to solve the objective function

The solution of objective function (10) is a multiple regularization constraints problem. In this paper, we use split Bregman iteration strategy^14,32,33^ to solve it.

The basic idea of split Bregman is introducing an auxiliary matrix **A** and modifying the objective function (10) to,

11 $$ \min f({\mathbf{Y}},{\mathbf{A}}) = ||{\mathbf{B}} - {\mathbf{GY}}||_{F}^{2} + \rho ||{\mathbf{Y}} - {\mathbf{Y}}_{{{\text{prior}}}} ||_{F}^{2} + \alpha ||{\mathbf{A}}||_{2,0} + \beta ||{\mathbf{A}} - {\mathbf{DY}}||_{F}^{2} $$

where *β* is an automatically adapting auxiliary parameter to control the similarity between the auxiliary matrix A and **DY**.

The objective function (11) does not impose strict equality constraints. The main idea behind split Bregman is to relax the equality in the initial iterations but enforce it toward the end (while the solution converges)^14,32^. In order to do so, the value of *β* is varied; usually, it is kept low initially, but as the solution converges, its value is progressively increased.

Then, Eq. (11) is solved iteratively by alternatively minimizing **A** and **Y** with two sub-problems. In each sub-problem, one variable is fixed with values obtained from the previous iteration.

Sub-problem 1: take the auxiliary matrix **A** fixed and estimate the model parameters **Y**. Hence, sub-problem 1 has the following sub-objective function,

12 $$ \min f({\mathbf{Y}}) = ||{\mathbf{B}} - {\mathbf{GY}}||_{F}^{2} + \rho ||{\mathbf{Y}} - {\mathbf{Y}}_{{{\text{prior}}}} ||_{2}^{2} + \beta ||{\mathbf{A}} - {\mathbf{DY}}||_{F}^{2} $$

It is a linear inversion problem constrained by strict quadratic regularization. Hence, the exact solution of Eq. (12) is,

13 $$ {\mathbf{Y}} = ({\mathbf{G}}^{T} {\mathbf{G}} + \beta {\mathbf{D}}^{T} {\mathbf{D}} + \rho {\mathbf{I}})^{ - 1} ({\mathbf{G}}^{T} {\mathbf{B}} + \beta {\mathbf{D}}^{T} {\mathbf{A}} + \rho {\mathbf{Y}}_{{{\text{prior}}}} ) $$

where **I** is the identity matrix.

One can see that Eq. (13) contains the inverse of a square symmetric matrix. In seismic inversion problem, these matrices are usually very big. Hence, we usually use some iterative algorithm to solve it, such as conjugate gradient algorithm.

Sub-problem 2: take the model parameters **Y** fixed and update the auxiliary matrix **A**. Hence, sub-problem 1 has the following sub-objective function,

14 $$ \min f({\mathbf{A}}) = \left\| {{\mathbf{DY}} - {\mathbf{A}}|} \right\|_{2}^{2} + \frac{\alpha }{\beta }\left\| {\mathbf{A}} \right\|_{2,0} $$

It is a linear inversion problem constrained by L_2,0_-norm regularization. We can use the row hard-thresholding algorithm to solve it^34^. Hence, the solution is,

15 $$ {\mathbf{a}}^{i} { = }\left\{ \begin{gathered} {\mathbf{0}},\begin{array}{*{20}c} {} & {||{\mathbf{x}}^{i} ||_{2}^{2} \le \alpha /\beta } \\ \end{array} \hfill \\ {\mathbf{x}}^{i} ,\begin{array}{*{20}c} {} \\ \end{array} ||{\mathbf{x}}^{i} ||_{2}^{2} > \alpha /\beta \hfill \\ \end{gathered} \right. $$

where **a**^*i*^ is the *i*th row of the auxiliary matrix **A**, **x**^*i*^ is the *i*th row of **DY**, **0** is a vector with all of elements equal to zero.

From the above, the specific steps of split-Bregman iteration algorithm to solve the objective function (10) are:

Input: multi-trace seismic data matrix **B**, wavelet convolution matrix **W**, difference operator matrix **D**, a priori impedance model *Ip*_prior_, regularization parameters *α* and *ρ*, initial auxiliary parameter *β*_0_.

Initialization: calculate the a priori logarithmic impedance **Y**_prior_ from *Ip*_prior_, set **A**_0_ = **O**, where **O** is a matrix with all of elements equal to zero, set *l* = 1.

Step 1: With **A** = **A**_*l*-1_ and *β* = *β*_*l*-1_, solve the sub-problem 1 to compute *Y*_*l*_.

Step 2: With **Y** = **Y**_*l*_, solve the sub-problem 2 to compute **A**_*l*_.

Step 3: If the following criterion for split-Bergman iteration is satisfied, stop the iteration,

16 $$ \frac{{||{\mathbf{Y}}_{l} - {\mathbf{Y}}_{l - 1} ||_{2}^{2} }}{{1 + ||{\mathbf{Y}}_{l} ||_{2}^{2} }} < \varepsilon $$

where *ε* is a tolerance value for split-Bergman iteration. If not, set,

17 $$ \beta_{l} = \tau \beta $$

where *τ* is an automatically increasing rate for *β*. As usual, its value is bigger than 1 and needs to be carefully selected.

Step 4: Set *l* = *l* + 1, and go back to step 1 for the iteration.

Having solved objective function (10), multiplied by two, a simple exponential transformation can give the final inverted impedance model^18^.

## Numerical tests and real data applications

### Numerical tests

**Figure 1:**
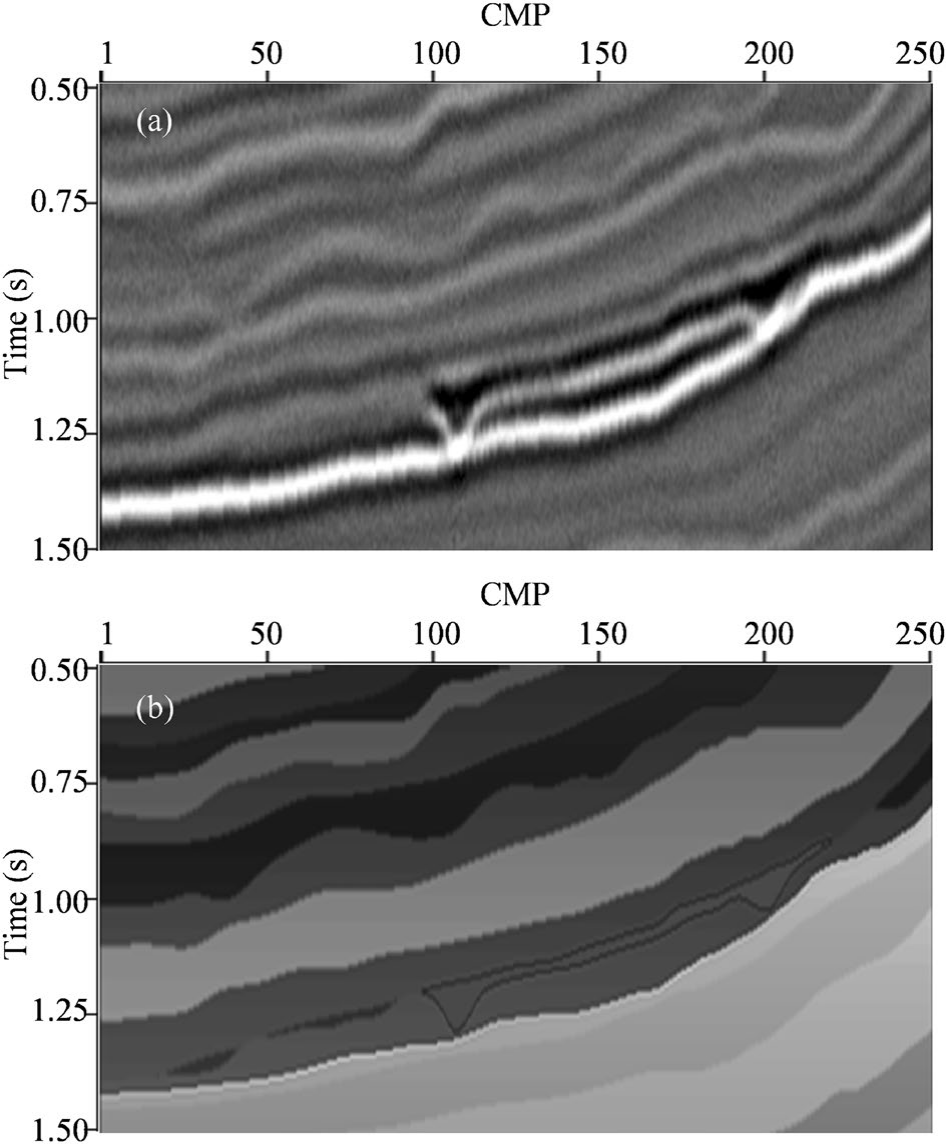
(**a**) Noise-tainted synthetic seismic data section. (**b**) The true 2D impedance model section.

First, we adopt a 2D synthetic seismic data section to test the feasibility of the proposed multi-trace impedance inversion with L_2,0_-norm regularization constraint (L_2,0_-MI). This synthetic seismic data section has been added into 15% Gaussian random noise with zero-mean. The noise-tainted synthetic seismic data section is shown in Fig. 1a. The corresponding true 2D impedance model section is shown in Fig. 1b. From the true impedance model, we can see that it contains thick layers, thin-bedded layers, and some lenticular-bodies. The stratigraphic changes in local areas are relatively continuous and stable.

**Figure 2:**
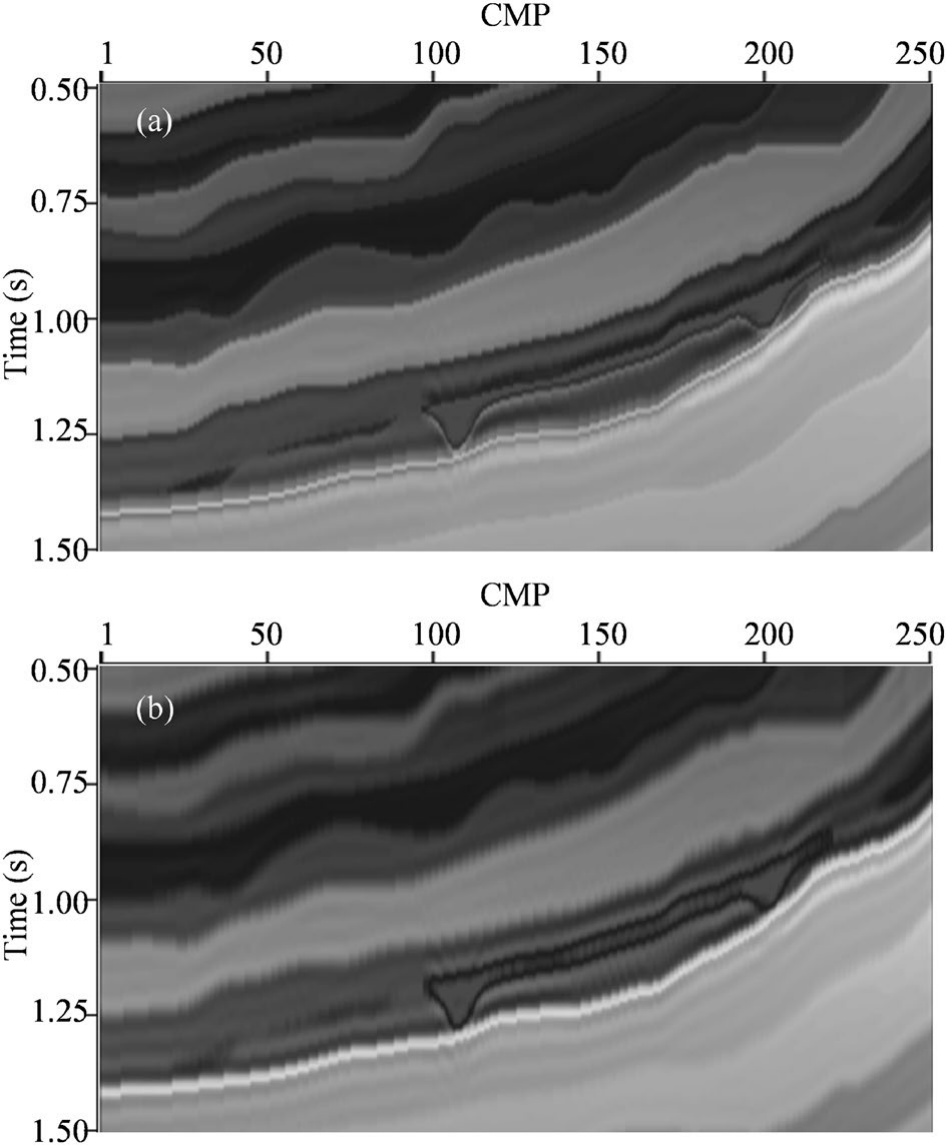
(**a**) The inverted impedance model section by L_2,0_-MI. (**b**) The inverted impedance model section by CSI.

The true 2D impedance model is smoothed through a low-pass filter (the threshold value is 15 Hz) to obtain a low-frequency trend model to serve as the a priori impedance model. Then, L_2,0_-MI is performed on the noise-tainted synthetic seismic data. The inverted impedance model by L_2,0_-MI is shown in Fig. 2a. From the comparison between the Figs. 1b and 2a, we can see that the inverted impedance model by L_2,0_-MI faithfully matches the true impedance model. The difference is very small. The inverted impedance model possesses a “blocky” structure and the vertical boundaries of different strata are very clear. It is due to the sparse regularization constraint effect in L_2,0_-norm. In addition, the lateral resolution and continuity is very well. The edges of lenticular-bodies are very clear. The lateral distribution characteristics of both layers are also very clear.

**Figure 3:**
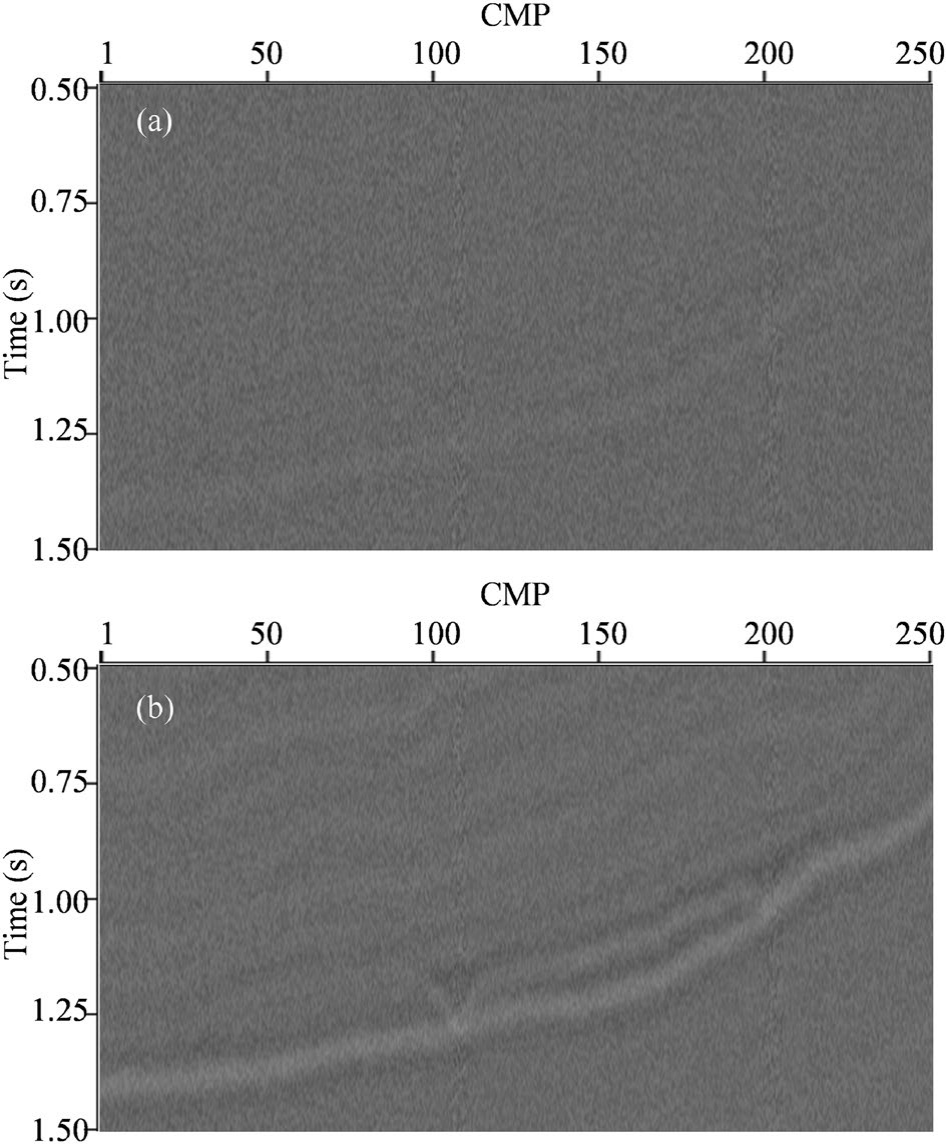
(**a**) The residuals between predicted data by L_2,0_-MI and noise-tainted synthetic seismic data. (**b**) The residuals between predicted data by CSI and noise-tainted synthetic seismic data.

To show superiority of L_2,0_-MI in lateral resolution and stability, the conventional sparse regularization constraint impedance inversion (CSI) is performed on the noise-tainted synthetic seismic data with the same low-frequency trend model as L_2,0_-MI to serve as the a priori impedance model. To fairly compare with L_2,0_-MI, a ω-x prediction filter has been included in the process of CSI to improve spatial continuity^35^. The inverted impedance model by CSI is shown in Fig. 2b. From the comparison between the Figs 1b and 2b, we can see that the inverted impedance model by CSI can also match the true impedance model. The inverted impedance model also possesses a “blocky” structure and the vertical boundaries of different strata are also very clear. It is due to the sparse regularization constraint effect. However, the lateral distribution characteristics in inverted impedance by CSI are less clear compared to inverted impedance by L_2,0_-MI. For example, the edges of lenticular-bodies are indistinct in Fig. 2b. It is because CSI only constrains inversion in the vertical direction through sparse regularization and does not use lateral regularization. Figure 3 shows the residuals between predicted data by two inverted impedance model and noise-tainted synthetic seismic data. We can see that the residual strength of L_2,0_-MI is visually smaller than CSI.

To quantificationally compare the quality of inverted impedance model from L_2,0_-MI and CSI, we calculate the relative errors (RE) of the above different inverted impedance model compared to the true impedance model. The REs are listed in Table 1. From Table 1, we can see that the REs of inverted impedance model by L_2,0_-MI are well below the REs of inverted impedance model by CSI.

**Table 1:** The REs of different inverted impedance model.

Methods	RE
CSI L_2,0_-MI	0.1083 0.0649

From the test results of 2D numerical model, we can see that, compared to the CSI, L_2,0_-MI can not only clearly estimate the vertical variation features, but also improve the lateral stability and resolution.

### Real data applications

**Figure 4:**
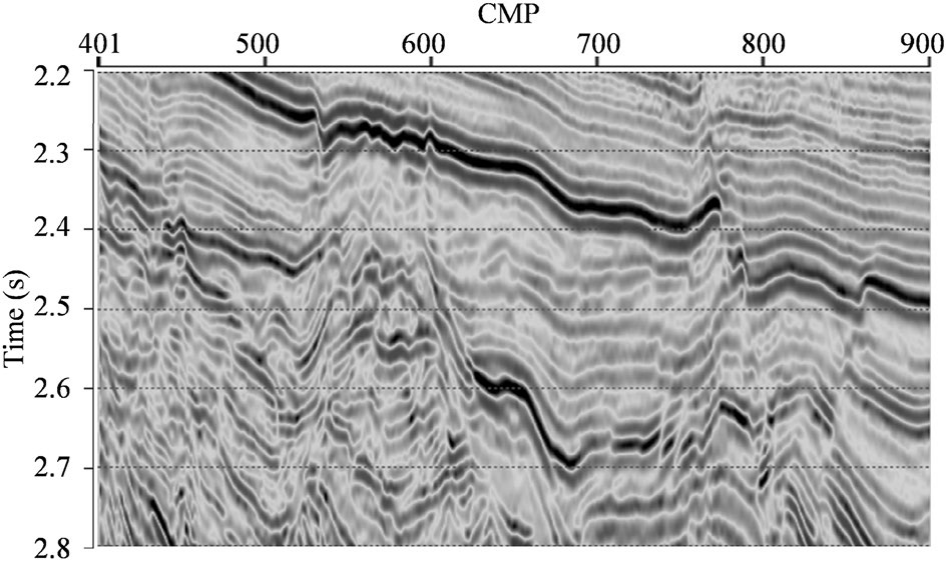
Real seismic data section.

Next, we adopt a real seismic data section from a work area in Eastern China to study the applicability of L_2,0_-MI in practice. Figure 4 shows this real seismic data section. Then, an interpolating impedance model is built by Kriging interpolation method with actual well logs in this work area under the constraint of seismic geologic horizon. After that, the 15 Hz low-pass filter is performed on the interpolation model to obtain a low-frequency trend model to serve as the a priori impedance model.

**Figure 5:**
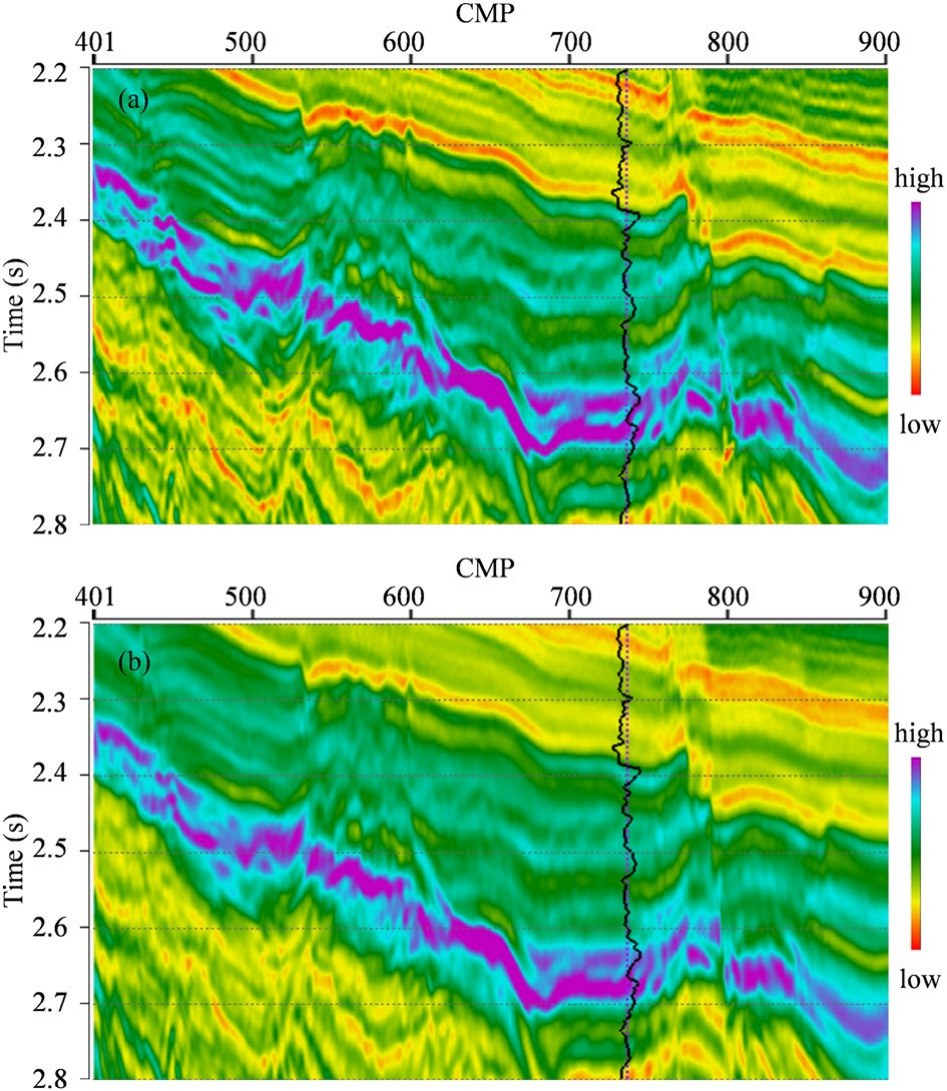
(**a**) Inverted impedance section by L_2,0_-MI. (**b**) Inverted impedance section by CSI.The black curve is the actual impedance well log.

L_2,0_-MI is performed on the real seismic data section. The inverted impedance section is shown in Fig. 5a. The actual impedance well log is overlaid in Fig. 5a for the comparison between the actual well log and the inverted impedance. From Fig. 5a, we can see that, the structural configurations and stratigraphic lateral distributions in the inverted impedance section match with the real seismic data section. The inverted impedances are well-matched with the actual impedance well log. The vertical boundaries of different strata are very clear, i.e. high vertical resolution. In lateral direction, both the transverse distribution characteristics of underground formations and the transverse interfaces of different faults are very clear. Hence, the inverted impedance section by L_2,0_-MI also has good lateral stability and resolution. Then, CSI is also performed on the real seismic data section with the same low-frequency trend model to serve as the a priori impedance model. The inverted impedance section by CSI is shown in Fig. 5b. As well, the actual impedance well log is also overlaid. From Fig. 5b, we can see that, the inverted impedance by CSI is also well-matched with the actual impedance well log. The structural configurations and stratigraphic lateral distributions in the inverted impedance section by CSI also match with the real seismic data section. And the vertical boundaries of different strata are also very clear. However, the lateral distribution characteristics and the interfaces of different faults in inverted impedance by CSI are less clear compared to inverted impedance by L_2,0_-MI.

**Figure 6:**
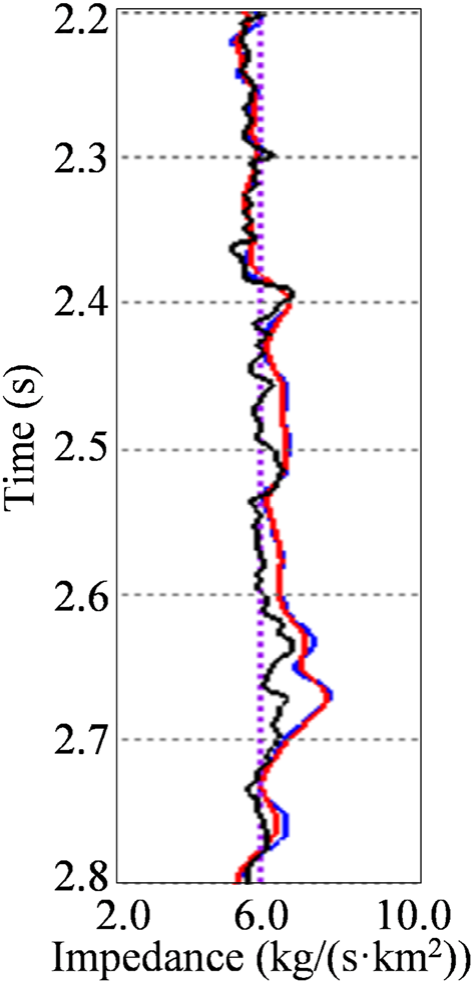
Comparison between actual impedance well log and two different inverted impedances at near-well trace. The black curve is the actual impedance well log, the blue curve is the inverted impedance by L2,0-MI, and the red curve is the inverted impedance by CSI.

Figure 6 further compares two inverted impedance by L_2,0_-MI and CSI at near-well trace with the actual impedance well log. The black curve is the actual impedance well log, the blue curve is the inverted impedance by L_2,0_-MI, and the red curve is the inverted impedance by CSI. We can see that, in some layers, the inversion results cannot coincide with the well log completely. It is because the scale of seismic data and well log data is different. The inverted impedance cannot coincide with the actual well log completely. The general variation trend and local relative variation characteristics of the two inverted impedances are in good agreement with the actual well log. Hence, we say that both two inverted impedances are really well-matched with the actual impedance well log. In addition, the vertical resolution of L_2,0_-MI is somewhat improved in some layers.

From the real seismic data applications, we can also see that, compared to the CSI, L_2,0_-MI can not only clearly estimate the vertical variation features of underground formations, but also improve the lateral stability and resolution.

## Discussions

From the main body of the “Methodology”Section, we can see that, there are some key factors to effect the quality of L_2,0_-MI. First, we think the lateral changes of underground formations are relatively stable in sedimentary strata, i.e., the strata have local lateral continuity. Hence, we need to partition the whole seismic data section into many small blocks. In these blocks, the local lateral continuity is much more likely to be satisfied. To avoid blocking artifacts at the connecting location, these blocks need to be overlapped. Hence, the block size and the overlap size need to be carefully selected and is controlled by the geologic features of the studied work area. Second, the two regularization parameters *α* and *ρ*, also greatly effects the inverted impedance. The inversion results are sensitive to different lateral regularization options. During the inversion procedure, the regularization parameters act as a tradeoff between the joint-sparsity and density of reflectivity series. In practice, the choice of the regularization parameters depends on the actual geological setting of underground formations and the aim of seismic inversion. If the underground formations are main thick and the lateral continuity is good, or the aim of inversion is to invert main stratigraphic sequence interface of underground formation, the value of *α* needs to be large. In this case, it is in fact equivalent to sparse-spike inversion with joint-sparse constraint. On the other hand, if the underground formations contain thin interbed, or the inversion is performed near the location of abrupt lateral changes, we need to, carefully choose the block size when partition the seismic data, and relatively decrease the value of *α*. In this paper, we adopt the quality control method to select the above parameters, i.e., block size, overlap size, two regularization parameters *α* and *ρ*. That is, adjust the parameters to be selected, get the inversion result for each value, and choose the one whose inversion result has the best match with the actual well log.

In general, when the post-stack inversion problem is solved in the multi-trace way rather than trace-by-trace, the computation resources will increase. In this paper, we use split Bregman algorithm to solve the objective function of L_2,0_-MI. The basic consideration of using this algorithm is that the iteration process is simple and the program implementation is relatively easy. In the framework of split Bregman algorithm, compared to CSI, the additional computation costs of L_2,0_-MI per iteration are just once calculation of row hard-thresholding. Hence, the computational time of L_2,0_-MI is just little longer than CSI.

Another factor that affects the computational cost and quality of L_2,0_-MI is auxiliary parameter *β* in split-Bergman iteration. This auxiliary parameter *β* is automatically adapted in iterations starting from a small initial value with a multiplier *τ* (i.e. automatically increasing rate). A smaller multiplier gives higher-quality inverted impedance, but at the cost of a longer computational time. Hence, the multiplier *τ* is used to balance the efficiency and performance. From a number of experiments, *τ* = 1.2 seems to be a good choice.

Here, L_2,0_-MI is dealt with at the framework of deterministic inversion. Usually, the uncertainty of deterministic inversion results can be assessed with a posterior covariance through a Bayesian point^9^. However, due to the joint sparse constraint represented by L_2,0_-norm, the posterior covariance cannot be analytically expressed. Besides deterministic inversion, the uncertainty can also be effectively assessed on the framework of stochastic inversion. It includes Bayesian inversion^3^ and iterative geostatistical inversion^4,36^. Stochastic inversion defines the inversion results as a probability distribution of model parameters. Hence, it can assess different sources’ uncertainties, such as original observed seismic data, well log data, or the a priori model and initial model^36,37^.

To perform uncertainty assessment in the process of L_2,0_-MI, an alternative way is to execute L_2,0_-MI on the framework of iterative geostatistical inversion. On that occasion, Eq. (10) can be used as the objective function of iterative geostatistical inversion. In iterative geostatistical inversion, the common used objective function base exclusively on the misfit or correlation coefficients between observed and predicted seismic data^38^. Azevedo and Soares have proposed a method to incorporate a priori model constraint into geostatistical inversion^38^. In fact, the a priori model constraint are common used in deterministic inversion. Hence, we can also incorporate the joint sparse constraint into the framework of iterative geostatistical inversion. But it can be a future research topic and is outside the scope of this paper.

## Conclusions

In this paper, L_2,0_-norm has been used as an joint-sparse regularization constraint to replace L_0_-norm in the conventional sparse regularization constraint impedance inversion and construct the joint-sparse regularization constrained objective function for multi-trace seismic impedance inversion. It is because the conventional sparse regularization constraint only considers the vertical sparsity, and does not consider the lateral variation feature and continuity of multi-trace impedance model. Whereas, L_2,0_-norm can not only measure the sparsity in the vertical direction, but also consider the lateral similarity and continuity of multi-trace impedance model to increase the lateral resolution and continuity. From the 2D numerical model tests and the real seismic data applications, we can see that, compared to the inverted impedance by CSI, the inverted impedance by L_2,0_-MI has higher lateral stability and resolution. Hence, compared to the CSI, L_2,0_-MI can not only clearly estimate the vertical variation features, but also improve the lateral stability and resolution.

## Data Availability

The datasets used during the current study are available from the corresponding author on reasonable request.
